# Carbapenem Restriction and its Effect on Bacterial Resistance in an Intensive Care unit of a Teaching Hospital 

**Published:** 2013

**Authors:** Mohammad Sistanizad, Mehran Kouchek, Mohammad Miri, Reza Goharani, Mehrdad Solouki, Ladan Ayazkhoo, Masoumeh Foroumand, Majid Mokhtari

**Affiliations:** a *Department of Clinical Pharmacy, Faculty of Pharmacy, Shahid Beheshti University of Medical Sciences, Tehran, Iran.*; b *Department of Critical Care, Imam Hussein Educational Hospital, Shahid Beheshti University of Medical Sciences, Tehran, Iran.*; c *Imam Hussein Educational Hospital, Shahid Beheshti University of Medical Sciences, Tehran, Iran. *

## Abstract

Development of antibiotic resistance in Intensive Care Units (ICUs) is a worldwide problem. The purpose of this study was to evaluate the effect of an antibiotic stewardship program (ASP) by carbapenems restriction on gram-negative antimicrobial resistance in ICU. The study was designed in a 21 bedded general ICU of a teaching hospital with two wings (one and two) in Tehran, Iran. Carbapenem prescription in ICU1 was restricted to only the culture proven multi-drug-resistant bacteria with the absence of sensitivity to other antimicrobial agents. Carbapenem had to be prescribed by a trained ICU physician with close consultation with infectious disease specialist and the clinical pharmacist posted in ICU. Post-prescription reviews and de-escalations were carried out by the same team on regular basis. Restriction policy was commenced in January 2011 in ICU1. All documented infections and resistance patterns of isolated pathogens were recorded in both ICUs during two periods of 6 months before and 9 months after restriction policy implementation. During this study bacterial growth was detected in 51.5% of 1601 samples. Carbapenem administration was decreased from 6.86 to 2.75 DDD/100 patients day (60% decreases) pre-restriction and post-restriction respectively. Significant increase in sensitivity of pseudomonas to imipenem was observed in ICU1 comparing with pre-restriction period six months post restriction (p = 0.000). Sensitivity of *Klebsiella *and Acinetobacter to imipenem did not change significantly during the study period. Conclusion: Our study demonstrated that restriction of carbapenems can increase sensitivity of *P. aeroginosa *to imipenem.

## Introduction

Antibiotic resistance is a major and increasing problem in the arena of infectious diseases as a whole and in the therapy of hospital infections in particular (1-3). The emergence of antimicrobial-resistant organisms is accelerating, and novel drug development has not kept up with its pace (4-6). Serious infections caused by bacteria that have become resistant to commonly used antibiotics have become a major global healthcare problem in the 21^st^ century (7). They not only are more severe and require longer and more complex treatments, but they are also significantly more expensive to treat (7-12). Similar problems are reported from other countries such as Iran focusing on ICU related complex microbiology and difficulties in their management (13-14). 

Although the need for new antimicrobials is increasing, development of such agents faces significant obstacles (14, 15). From 1998 to 2002, FDA approval of new antibacterial agents decreased by 56%, compared with the period from 1983 to 1987 (4). A numberof factors like high pharmaceutical research and development costs and the aging of populationmake antimicrobial agents less economicallyattractive targets for development than other drug classes (16). 

Given the association between antimicrobial use and the selection of resistant pathogens, the frequency of inappropriate antimicrobial use is often applied as a surrogate marker for the avoidable impact on antimicrobial resistance (17). Solutions currently argue decreasing the use of antibiotics for human infections that are self-limited or likely to have been caused by viruses and rational use of antimicrobial agents in hospitalized patients (18-20). Betterloyalty to infection control guidelines has become a national priority for preventing health care–acquired infection and anti-microbial-resistant infections (21, 22).

Hospital antimicrobial stewardship programs (ASPs), focus on the development of effective hospital-based program which includes appropriate selection, dosing, route, and duration of antimicrobial therapy commonly using formulary restrictions and by requiring preauthorization (17). Since 2000, high rates of resistance of *Pseudomonas aeruginosa*, *Acinetobacter baumannii *and *Klebsiella pneumoniae *to third-generation cephalosporins (represented by ceftazidime) as well as to fluoroquinolones have been documented in the ICU, leading to increased use of carbapenems and subsequently to the emergence of resistance to carbapenems (23). As carbapenem resistance among gram negative organisms is increasing because of increased use, these antimicrobial agents are restricted in some hospitals for treatment of gram-negative bacterial infections resistant to first-line drugs (24-29). 

The objectives of this study are to examine how implementation of an institutional carbapenem stewardship program affects P. aeruginosa, A. baumannii K. pneumoniae and E. coli susceptibility and antibiotic use in the ICU setting. 

## Experimental

*Hospital setting*

This study was designed in a general ICU of a 600-bed teaching hospital with two wings of 1 and 2 in Tehran, Iran. BothICUs have dedicated unit-based critical care physicians and clinical pharmacy specialists. In addition, clinical staffs verify orders on all shifts and are instructed to intervene on restricted medication orders if used outside of criteria. They also make sure that the teams are following the antibiotic restriction protocol.

*Antibiotic stewardship program*

This interventional cohort studywas structured in two phases. Phase I, preparation/information, from September to November 2010, was designed to improve physicians’ knowledge about and attitudes toward carbapenem use. This was followed by phase II, the intervention period, which started in January 2011. Carbapenems (imipenem and meropenem) were restricted in ICU 1 to culture proven multi drug resistant bacteria with the absence of sensitivity to other antimicrobials agents. Carbapenem could be prescribed by trained ICU physicians with close consultations with infectious disease physicians and clinical pharmacist posted in ICU. Daily rounds took place by the intensivist and clinical pharmacists with infectious disease consultations available on request. Post-prescription reviews including adjustments and de-escalations were carried out by the same team on regular basis. Investigators communicated with the physicians responsible when necessary. Disagreements between investigators were addressed by discussions and review of published guidelines. Antibiotic consumption;amikacin, imipenem, piperacillin/tazobactam, gentamicin and ciprofloxacin was estimated as number of Defined Daily Doses (DDD) per 100 ICU patients-days per WHO collaborating center for drug statistics methodology (http://www.whocc.no/atc_ddd_index) in both phases of the study.

Infection control policies remained unchanged during the whole study period with no change in patient mix and no antibiotic formulary restriction. Antimicrobials replacing Imipenem and meropenem as empirical therapy during the intervention period included mostly cefepime and piperacillin/tazobactam.

*Microbiology*

All isolates from any source including blood, urine, sputum, catheter and wound were identified by standard microbiological methods, their susceptibilities have been interpreted by disk diffusion method according to Clinical Laboratory Standards Institute (CLSI) guidelines (30). Imipenem resistance was evaluated as it was the carbapenem used most frequently in our ICU.Results are expressed in three categories (S: sensitive, I: intermediate, R: resistance).

*Data collection*

All documented gram negative infections with P. aeruginosa, Acinetobacter, *Klebsiella *and *E. Coli*, and resistance patterns of isolated pathogens to imipenem, amikacin, gentamicin, cefepim, piperacillin and ciprofloxacin were recorded in two periods, 6 months before, 3 months, 6 months and 9 months after implementation of the restriction policy in both ICUs. 

*Statistical analysis*

Data were analyzed using the SPSS version 16.Chi2test was performed and a P value of <0.05 was accepted as significant.

## Results

*Patients*

During our study, a total of 1601 samples were sent from both ICUs to microbiology laboratories for culture and sensitivity, 607 of which belonged to the pre-restriction and the remainingto the post-restriction period. Bacterial growth was seen in 51.5 percent of samples. 

*Antibiotic consumption*

The amount of carbapenem administration decreased by 64% from 87 vials/month in pre-restriction period to 31.9 vials/month during the post-restriction phase (p-value= 0.02). Total antibiotic use did not change significantly (p-value = 0.904). Results of antibiotics consumption based on DDD/100 PD are shown in table 1.

**Table 1:** Consumption of antibiotics before and after implementation of restriction policy based on DDD/100 patients day

**Antibiotic**	**ICU-1**				**ICU-2**		
	**pre-restriction**	**post-restriction**		**% difference**	**pre-restriction**	**post-restriction**	**% difference**
**Amikacin**	7.97		8.02	0.63	5.4	7.87	45.74
**Imipenem**	6.86		2.75	-59.91	11.96	6.16	-48.49
**piperacillin/ tazobactam**	4.82		5.56	15.35	5.99	2.85	-52.42
**Gentamicin**	9.93		17.02	71.40	1.3	1.50	15.38
**Ciprofloxacin**	20.91		19.98	-4.45	18.06	25.48	41.09
**total**	50.49		53.32	5.61	42.71	43.85	2.67

*Bacterial susceptibilities and colonization data*

**Figure 1:**
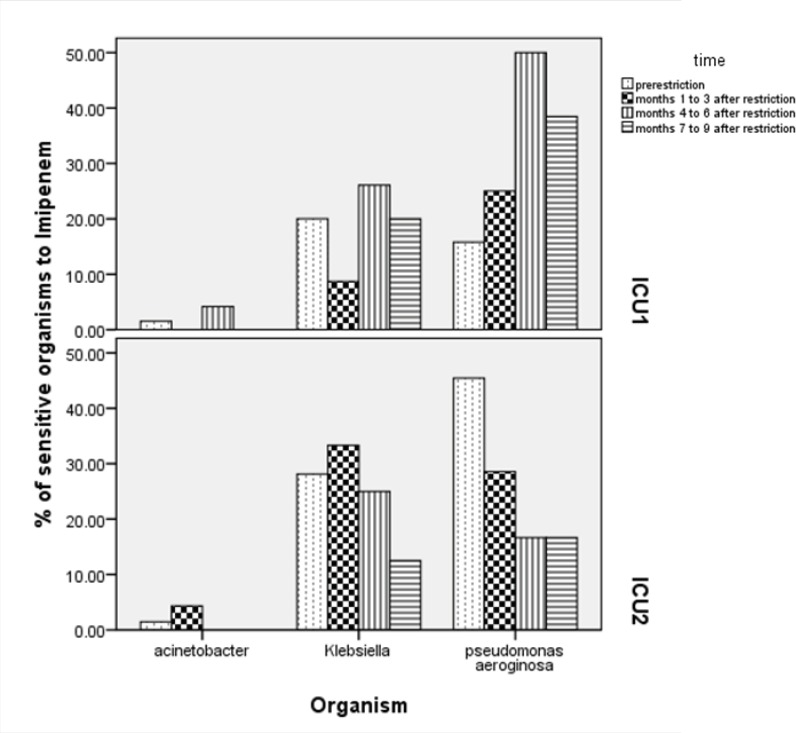
Sensitivity of leading microorganisms in ICU to imipenem 6months before and 1-3, 4-6 and 7-9 months after implementation of restriction policy

Analysis of results in 3 months period (Chi^2^=5.028, p-value= 0.25), 6 months (Chi^2^=29.94, p-value = < 0.01) and 9months (Chi^2^=21.35, p-value= < 0.01) showed increase in sensitivity of pseudomonas to imipenem 6 months after implementation of restriction policy. Changes in sensitivity of microorganisms to imipenem in 2 ICUs in periods of 3 monthscompared to pre-restriction period are shown in Figure 1.

## Discussion

Before implementation of restriction policy, the susceptibility of four common gram-negative microorganisms in the ICU (P.aeroginosa, A.baumannii, K.pneumoniae and E. coli) to imipenem, ciprofloxacin, amikacin, gentamicin and piperacillin was extremely low,although susceptibility of these microorganisms to imipenem was reportedly more prominent in prior studies (31).

The sensitivity of P*. aeroginosa *to amikacin, ciprofloxacin, gentamicin and imipenem increased significantly after carbapenem restriction (Table 2 and Figure1).

**Table 2:** Sensitivity of main gram negative microorganisms to antibiotics before and after implementation of carbapenem restriction policy

**Microorganism**	**Antibiotic**	**ICU 1**			**ICU 2 **		p-value
		pre-restriction	post-restriction	pre-restriction		post-restriction	
P. aeroginosa	amikacin	21.05	45.83	33.33		30.43	<0.01
P. aeroginosa	ciprofloxacin	36.84	52.63	50.00		33.33	0.01
P. aeroginosa	piperacillin	21.05	29.17	25.00		22.22	0.27
P. aeroginosa	gentamicin	22.22	30.43	41.67		21.74	0.01
P. aeroginosa	imipenem	15.79	38.10	45.45		19.05	<0.01
A. baumannii	amikacin	1.54	19.81	3.08		12.78	0.62
A. baumannii	ciprofloxacin	1.56	0.99	1.47		0.00	-
A. baumannii	piperacillin	0.00	1.08	0.00		0.00	-
A. baumannii	gentamicin	3.28	10.10	0.00		24.44	0.12
A. baumannii	imipenem	1.52	1.05	1.47		0.81	0.71
K. pneumoniae	amikacin	0.00	37.50	8.82		20.00	<0.01
K. pneumoniae	ciprofloxacin	11.76	10.07	11.76		8.57	0.09
K. pneumoniae	piperacillin	5.88	13.64	3.45		27.27	0.07
K. pneumoniae	gentamicin	18.75	13.70	21.88		17.14	0.92
K. pneumoniae	imipenem	20.00	18.31	28.13		21.21	0.68
E.coli	amikacin	11.11	50.00	30.00		56.00	0.12
E.coli	ciprofloxacin	31.25	20.00	40.00		37.50	0.29
E.coli	piperacillin	20.00	13.04	37.50		25.00	0.98
E.coli	gentamicin	60.00	47.62	100.00		62.50	0.39
E.coli	imipenem	47.06	40.90	40.00		43.48	0.50

The results of our study also indicatedsignificant decrease in carbapenem use in both ICUs despite implementation of restriction policy in ICU-1. This could be due to the similarities in physicians’ practice in both ICUs.

Decrease in antibiotic consumption after implementation of restriction policies have been shown in several studies (32, 33). Bantar *et al*. (33) developed an intervention program to optimize hospitalantibacterial use and demonstrated a statistically significantdecrease in carbapenem use over a 2-year period (from 13.5 to6.2 DDD/1,000 PD; p= 0.03).

We showed significant increase in the susceptibilities of P. *aeroginosa *to *amikacin*, ciprofloxacin, gentamicin, and imipenem and K. *pneumoniaetoamikacin*, ciprofloxacin and piperacillinfollowing 9-months restriction of empirical use of imipenem. 

Results similar to our study have been reported in multiple studies after ASP implementation (28, 32-35). Bantar *et al*. (33) showed asignificant decrease in the amount of imipenem-resistant P.aeruginosa isolates, from 19% to 0% after significant decrease in carbapenem use. Pakyz *et al. *(28) evaluated the relationship between carbapenem restriction and the volume ofcarbapenem use and both the incidence rate and proportion of carbapenem-resistant *Pseudomonas aeruginosaisolates *from 2002 through 2006 in a retrospective, multicenter investigation among a group ofacademic health centers. A survey inquired about restriction policies for antibiotics, including carbapenems. 8 (36%) of 22 hospitals that restricted carbapenems use significantly (p = 0.04) and reported lowerincidence rates of carbapenem-resistant P. *aeruginosa *(p = 0.01) for all study years. They concluded that restriction of carbapenems is associated with both lower use andlower incidence rates of carbapenem resistance in P. *aeruginosa.*

White and colleagues(36) studied the effect of an antimicrobial control program onantimicrobial expenditures and susceptibilities. Monthly expendituresfor imipenem during the program decreased by 40% and the proportion of P. *aeruginosa *isolates susceptible toimipenem increased significantly, from 83% to 95% for inpatientsand from 65% to 83% for intensive care unit patients.Martin *et al*. (37) described an antimicrobial stewardshipprogram that restricted the use of carbapenems and found improvement in carbapenem susceptibilities for P. *aeruginosaover *5 years, from 86% for the first year of the study to 91%in the last year. Carbapenem use was variable throughoutthe study period and actually increased from the first studyyear to the final year. Ong et al. in a prospective cohort study showed thatmeropenem consumptionin ICU patientswith P. *aeruginosa *was associated with antibioticresistance development to meropenem. The association was stronger formeropenem than for other antibiotics. These findings indicate that an increase in carbapenem use as a result of theglobal emergence of Gram-negative bacteriaproducing extended-spectrumbeta-lactamases creates a serious risk forrapid emergence of carbapenem resistance among P. aeruginosa. Therefor antibiotic stewardship to optimize carbapenems use (*i.e*., to minimize its unnecessary administration) is recommended (35). Ntagiopoulos et al. indicated a significant increase in the susceptibilities of the three most important Gram-negative pathogens to ciprofloxacin following an 18-month restriction on the empirical use of fluoroquinolones and ceftazidime in a general ICU (23).

There are several limitations of our study. We were not able to investigate whether restrictive use of antibiotics was associated with any mortality benefit or had any impact on ICU cost. Also our study period after ASP implementation may have not been long enough to see the changes in antimicrobial resistance, especially in Acinetobacter species. Lack of information regarding MIC of antimicrobials could be another limitation of our study.

## Conclusions

Development of bacterial resistance has been deemed as one of the most problematic outcome of inappropriate antimicrobials use. This phenomenon has adversely impacted the care of infected patients particularly those with critical illness by increasingmicrobial resistance, morbidity, mortality and cost. Appropriate and judicious antimicrobial use guided by an ASP can be associated with significant multifaceted benefits in critically ill patients. 
